# Some Mental and Moral Aspects of the Strike

**Published:** 1912-03-23

**Authors:** 


### The Hospital

The Workers' Newspaper of Administrative Medicine and Institutional Life, Administration, National Insurance and Health.

No. 1341, Vol. LI. SATURDAY, MARCH 23, 1912. PRICE ONE PENNY.

### SOME MENTAL AND MORAL ASPECTS OF THE STRIKE.

Having closely studied the labour questions and the conditions of the people in this country, the United States, and Europe, we are impressed with the feeling that the politicians of this country are displaying an absence of statesmanship and business ability, and a want of apprehension of the moral and mental aspects of the coal strike and what lies behind it. The Churches, too, or many of them, have failed to grasp the situation and to take their share in adjusting the present social upheaval which, wisely handled, is capable of making England the brightest and happiest place for the worker the world can offer. We have nothing to do with politics in The Hospital, but we have much to do with the physical well-being and mental and moral soundness of the inhabitants of these islands. Up to the present time the workmen have displayed commendable self-command and resolution, especially those amongst them who are not directly concerned in the coal strike, but whose wives and children are suffering much regrettable hardship from it. The first danger to avoid is everything which may tend to increase such suffering amongst these innocent outside workers. Absence of food, of warmth, of wages to maintain the home, with their attendant discomforts and miseries, must quickly produce illness and suffering, whilst the holding up of supplies must speedily result in a breakdown of the sanitary provision whereby the hygienic condition of towns and the general health of the community are maintained in these islands at the present high standard of efficiency. The first thing therefore is undoubtedly to secure the prompt ending of the present strike.

If guarantees are forthcoming that the legislation proposed by the Government will end the strike at once, but not otherwise, the temporary nature of the remedy may be accepted, providing the Government can satisfy Parliament and the nation that they will not rest until further legislation gives to the nation as a whole, and to the working-classes as individuals, the security without which the trade and prosperity of these islands must speedily be lost. No statesmen, whatever may be their party politics, can at a juncture like the present rest content with temporary legislation sufficient only to tide over the present difficulties, until those in office are succeeded by a new Government. No Opposition and no individual member of Parliament, possessing the elements of statesmanship who are worthy of the confidence of the electors, can vote for temporary legislation which neither contains safeguards whereby the new Act can be enforced, impartially, upon owners and workmen, nor guarantees that it will be followed promptly by further legislation which will place the labour question as far as these islands are concerned upon a lasting basis, satisfactory to all reasonable people possessed of common-sense, be they workmen, or employers, or private citizens. The need of the moment is statesmanship, and the danger of the moment is that an absence of statesmanship may result in the spread of the corybantic absurdities, due largely to a loss of mental balance in the individual, such as that displayed by certain irreconcilable suffragettes.

The mental and moral aspects of the present labour crisis lie at the root of the questions involved. The prosperity and wise government of Great Britain are great assets to the well-being of all peoples throughout the world. We are convinced, from an intimate personal knowledge of the British working-men, that they are on the whole a reasonable and prudent community, who, as individuals, are in overwhelming numbers worthy of the fullest confidence of all classes. Politicians have lost their sense of perspective. In recent years their minds have become so cramped and inelastic that they fail to see that narrow party aims and party ambitions must mean in the end the destruction of England. In the same way the working-class leaders, and especially those responsible for the policy of trade-unionism in this country, have enforced remorselessly the destruction of free labour, so that the best and most competent workmen are deliberately handicapped in their work, harried and worried to death, and compelled to withhold the fulness and best of their labour by the enforcement of rules the aim and object of which is to encourage the "Weary Willies" of the labour market and its inefficients at the expense of the competent and self-respecting workman. Thus the proceedings in Parliament in recent years have exhibited politicians, as the action of trades-unionism has exhibited the leaders of this movement, as wanting in the higher attributes which a standard of personal conduct gives to every thinking and capable man and woman everywhere. Personally politicians, trade-union leaders, and many religious teachers who put politics before everything have ceased, apparently, to take any count of morality in shaping their courses of action, and so have become a grave danger to England and its continuance as a nation. This failure to give due weight to morality may be traced back to a diminution of mental power, owing to sentimental emotionalism or a loss of balance in appraising values in the individual.

The remedies for these grave dangers are not far to seek. The whole nation is willing, we believe, to provide every English workman with a minimum wage which represents a sum equal to the maintenance of a comfortable home for all workers under present conditions. The public are prepared to take their proper share in providing the extra cost of this minimum wage on condition that the working-classes will unite in re-establishing free labour throughout these islands upon a basis which will enable every workman to receive the full value of his earning-power based upon his technical competence, whatever that earning-power may be. All the shackles and chains with which the British workman is at present bound by trades-unionism must be removed, and if in the result the "Weary Willies" and other decadents, being incompetent workmen, cannot earn a minimum wage, then all such people must be treated as a class apart. Later, as we are prepared to show, they can be readily dealt with upon equitable lines, which will free the nation from the further domination of a class of individuals, who have grown up amongst us through causes, which it must be the duty of our statesmen and religious teachers promptly and effectually to remove.

